# A 2024 Update on Growth Hormone Deficiency Syndrome in Adults: From Guidelines to Real Life

**DOI:** 10.3390/jcm13206079

**Published:** 2024-10-12

**Authors:** Luigi Simone Aversa, Daniela Cuboni, Silvia Grottoli, Ezio Ghigo, Valentina Gasco

**Affiliations:** Division of Endocrinology, Diabetes and Metabolism, Department of Medical Sciences, University of Turin, 10126 Turin, Italy; luigisimone.aversa@unito.it (L.S.A.); daniela.cuboni@unito.it (D.C.); silvia.grottoli@unito.it (S.G.); ezio.ghigo@unito.it (E.G.)

**Keywords:** growth hormone deficiency, adult, diagnosis, stimulation tests, hormone replacement therapy, treatment protocols, clinical guidelines

## Abstract

**Background:** Adult growth hormone deficiency (GHD) has been recognized since the late 1980s. The clinical manifestations of adult GHD are often nonspecific, and diagnosis relies on GH stimulation tests, which are intricate, costly, time-consuming, and may carry the risk of adverse effects. Diagnosis is further complicated by factors like age, sex, and BMI, which affect GH response during testing. Therefore, GH replacement therapy remains challenging, requiring careful individualized evaluation of risks and benefits. The aim of this review is to provide an update on diagnosing and treating adult GHD, addressing current limitations and challenges based on recent studies. **Methods:** We conducted a comprehensive review of the literature regarding the diagnosis and management of adult GHD by searching PubMed and EMBASE. Only articles in English were included, and searches were conducted up to August 2024. **Results:** A review of guidelines and literature up to 2024 highlights the significant heterogeneity in the data and reveals various protocols for managing GHD, covering both diagnostic and therapeutic approaches. **Conclusions:** Despite diagnostic and treatment advances, managing adult GHD remains challenging due to variable presentation and the need for personalized GH therapy. Future efforts should aim to improve and standardize diagnostic and treatment protocols.

## 1. Introduction

Adult growth hormone deficiency (GHD) occurs due to a reduced secretion of growth hormone (GH) from the anterior pituitary gland. Adult GHD can manifest as either childhood-onset GHD (CO-GHD) or adulthood-onset GHD (AO-GHD) and may occur as an isolated GHD or in conjunction with multiple pituitary hormone deficiencies [1,2,3,4]. The most common cause of CO-GHD is idiopathic, although other causes include congenital factors (e.g., genetic abnormalities), brain structural defects (e.g., empty sella syndrome, septo-optic dysplasia, and midline facial defects), and acquired factors (e.g., perinatal insults, craniopharyngioma, germinomas, and pituitary adenomas) [1,2,3,4,5,6]. In contrast, AO-GHD is most frequently acquired due to hypothalamic-pituitary tumors and/or their treatment [1,2,3,4,5,6]. Additionally, especially in the past decade, there has been a growing body of research identifying non-tumoral causes of GHD that were previously unrecognized, including traumatic brain injury, subarachnoid bleeding, ischemic stroke, and infections of the central nervous system [1,2,3,4,5]. As expected from the ubiquitous distribution of GH and insulin-like growth factor I (IGF-I) receptors, GHD is characterized by several signs and symptoms [7,8]. Signs and symptoms characterizing the adult GHD syndrome are changes in body composition, metabolic derangement, cardiac dysfunction, decreased muscle strength and exercise capacity, decreased bone mineral density (BMD), and impaired quality of life (QoL) [1,2,3,4,7,8]. None of these signs and symptoms are specific, but when combined with reduced GH release, they constitute a well-defined clinical entity known as adult GHD syndrome [7]. Recent studies have shown increased mortality in patients with hypopituitarism [9,10,11,12,13,14], especially in women and in younger patients [13,14,15]. While not definitively proven, it is probable that GHD itself is a contributing factor to the elevated rates of cardiovascular morbidity and mortality found in individuals with various pituitary disorders, in contrast to the general population [16,17,18]. However, other factors such as under- [10] or over-treatment [12,19] of glucocorticoid replacement therapy for central adrenal deficiency, as well as the underlying etiology of the hypothalamic-pituitary disease, are also significant contributors [16,17,20,21].

In a patient where adult GHD is suspected, it is crucial to establish the diagnosis before considering replacement therapy with recombinant human GH (rhGH).

The currently available guidelines clearly define the appropriate clinical context in which to investigate the potential presence of GHD and identify the categories of patients who should be considered for evaluation to diagnose GHD [1,2,3,4]. However, some still confuse real adult GHD (i.e., patients with a reduced GH response to stimulating tests due to acquired or genetic causes) with the natural decline in endogenous GH secretion that occurs with aging, leading to the continued inappropriate and unapproved use of GH replacement therapy for nonmedical purposes (e.g., in sports and anti-aging) [3].

Over the past 30 years, evidence has accumulated demonstrating the beneficial effects of rhGH replacement therapy in reversing many, though not all, of the metabolic abnormalities associated with this condition [22,23,24,25,26,27,28,29,30,31,32,33,34,35,36,37,38]. Nevertheless, controversy persists regarding the appropriate use of rhGH therapy in adults with GHD as recently demonstrated by an audit sponsored by the European Society of Endocrinology (ESE) [39]. This is largely due to factors such as the high cost of therapy, the burden of daily injections for some patients and caregivers, a lack of awareness among certain clinicians about the indications and benefits of rhGH in adults, difficulties in safely conducting GH-stimulation tests in clinical settings, and concerns about potential adverse effects after long-term therapy.

To summarize and update the current guidelines on adult GHD, we conducted a comprehensive review of the literature on the topic. In particular, our review aimed to highlight the still controversial issues in the management of adult GHD syndrome, such as which tests should be used for diagnosis, which cut-offs to apply, laboratory challenges in measuring GH and IGF-I, and uncertainties regarding the long-term effectiveness of therapy due to the lack of data on strong endpoints.

This document provides an update on the management of adult GHD, moving from diagnosis to treatment, and discussing its limitations and challenges in light of the most recent studies.

## 2. Methods

We conducted a comprehensive review of the literature regarding the diagnosis and management of adult GHD by searching PubMed and EMBASE. The search terms used included “(GH OR growth hormone) deficiency diagnosis”, “(GH OR growth hormone) deficiency management”, and “(GH OR growth hormone) deficiency (therapy OR treatment)”. No limitations were applied to maximize the sensitivity of the search. A filter for studies involving adult human subjects was used. We also examined the reference lists of identified studies and key review articles, including previously published reviews. Only articles in English were included, and searches were conducted up to August 2024.

## 3. Update on Adult GHD Diagnosing

### 3.1. Who and How to Test

The clinical syndrome resulting from GHD in adults is characterized by alterations in body composition, including increased visceral adiposity, decreased lean body mass, and reduced muscle performance, along with insulin resistance, impaired glucose metabolism, and dyslipidemia, leading to an increased cardiovascular risk. Additionally, there is a higher incidence of osteoporosis and fractures, contributing to an overall reduction in QoL [1,2,3,7,8]. It is evident that the absence of pathognomonic signs, as well as the frequent occurrence of these symptoms in the general population, makes the diagnosis of adult GHD challenging based solely on clinical presentation. In this context, identifying the ‘appropriate clinical setting’ in which to investigate GHD becomes crucial.

The main guidelines recommend considering the possibility of GHD in adults in the following cases [1,2,3,4]:

- A history of childhood-onset GHD.
- Patients with documented structural hypothalamic-pituitary disease (particularly expansive lesions and the outcomes of their treatment, whether surgical or radiotherapeutic; traumatic brain injuries; infiltrative diseases; ischemic events and subarachnoid hemorrhages; empty sella syndrome).
- Patients with diagnoses of other pituitary hormone deficiencies, such as secondary hypothyroidism, hypocortisolism, or hypogonadism.

Even when the appropriate clinical and medical history context is established, biochemical evaluation remains complex, and several key factors must be considered [3]:

- GH is secreted by the pituitary gland in a pulsatile pattern, influenced by factors such as sex, age, and body mass index (BMI).
- Serum levels of IGF-I can decrease due to protein or caloric malnutrition, poorly controlled diabetes mellitus (DM), chronic diseases, renal failure, and liver cirrhosis.
- There is a physiological age-related decline in GH levels, which must be distinguished from pathological GHD, usually caused by an identifiable etiology.

Therefore, random serum GH and IGF-I measurements are not reliable, and most patients require GH stimulation tests for accurate assessment.

Regarding the diagnostic approach, we will review the guidelines issued by the Growth Hormone Research Society (GHRS) in 1997 and updated in 2007 [1], the Endocrine Society (ES) in 2011 [2] and 2016 [4], and the American Association of Clinical Endocrinologists and American College of Endocrinology (AACE) in 2019 [3].

First and foremost, guidelines agree that diagnostic tests for confirming GHD should only be conducted if there is an intention to treat the deficiency [1,2,3]. Consistently, diagnostic investigations can be avoided in patients who have absolute contraindications to starting rhGH therapy.

The GHD diagnosis is made through the administration of a GH stimulation test [1,2,3]. Several tests are available, but the guidelines recognize:

- Insulin tolerance test (ITT) [1,2,3];
- GHRH + arginine test [1,2];
- GHRH combined with GHRP-6 [1];
- Glucagon stimulation test [1,2,3];
- Macimorelin test [1].

For the specific characteristics of each test, refer to Table 1.

The use of clonidine, L-DOPA, and arginine tests is no longer recommended by the guidelines in the diagnosis of adult GHD. However, it is noteworthy that in the audit on the management of GHD sponsored by the ESE, it was found that, even recently, 7% of cases used stimulation tests that are no longer considered valid by current guidelines for diagnosis. This finding is even more striking when considering that the glucagon test (GST), one of the tests recognized as valid for the diagnosis of adult GHD by all guidelines, was used in a comparable percentage of cases (6%) [39].

Historically, ITT has been accepted as the gold standard; however, it has several limitations: it is a laborious test with numerous contraindications, is unpleasant for the patient, and presents potential side effects. Consequently, its use has gradually decreased, and research has focused on identifying alternative tests. A viable alternative is the GHRH + arginine test, although its widespread application is limited due to the poor availability of recombinant GHRH, which has not been available in the United States since July 2008. The GST has become one of the most frequently used tests due to its numerous advantages, including good availability, reproducibility, safety, minimal contraindications, and lack of influence from GHD etiology on the response. Its main disadvantage is the lengthy duration (3 to 4 h), requiring multiple blood draws and intramuscular administration of the drug, often accompanied by gastrointestinal side effects [3]. With the approval of the oral Macimorelin test in 2017, its advantages in terms of diagnostic accuracy (efficacy, reproducibility, sensitivity, and specificity comparable to ITT and GHRH + arginine tests), tolerability and practicality (short duration with only three or four blood draws) suggest it will become increasingly utilized over time [3]; however, to date, the cost of Macimorelin remains a potential limitation to its widespread use.

In general, a single abnormal test is sufficient to diagnose GHD in adults. Nevertheless, the number of GH stimulation tests required in transition patients with childhood isolated GHD or suspected hypothalamic GHD depends on the clinician’s level of suspicion. If suspicion is high, a single GH stimulation test should be performed, while, if suspicion is low, two different GH stimulation tests should be conducted, using appropriate peak GH cut-off points [1,3]. However, in a recent Italian consensus statement on the appropriate management of GHD during the age of transition, the expert panel disagreed on the number of retests based on degree of clinical suspicion [54].

Although, as previously mentioned, the diagnosis of GHD is based on the performance of stimulation tests of the somatotropic axis, the guidelines recognize certain conditions in which the presence/persistence of GHD can be assumed:

- Patients with organic hypothalamic-pituitary who have three or more pituitary hormone deficiencies and low serum IGF-I levels (<−2 SDS);
- Patients with CO-GHD due to genetic defects affecting the hypothalamic-pituitary axes;
- Patients with CO-GHD due to hypothalamic-pituitary structural brain defects.

The pre-test probability that a patient with organic hypothalamic-pituitary disease has GHD increases with the number of accompanying pituitary hormone deficits. For instance, there is a 45% probability of GHD in the absence of other deficits. Patients with three or more pituitary hormone deficits and IGF-I levels below reference values have a probability of over 97% of also having GHD. Moreover, the presence of three or more pituitary hormone deficiencies, with or without low IGF-I levels, carries a probability of GHD greater than 95% [55]. Therefore, according to the guidelines, patients with multiple pituitary hormone deficiencies, defined as three or more other pituitary hormone deficits, and serum IGF-I levels below −2 SDS [1,2,3] do not require GH stimulation tests for the diagnosis of GHD. Conversely, in patients with fewer than two pituitary hormone deficits, low serum IGF-I levels (<−2 SDS) alone are not sufficient to diagnose GHD in adults, and GH stimulation testing should be performed to confirm the diagnosis [3].

### 3.2. Caveats in Performing and Interpreting the Different Diagnostic Tests

It is necessary to consider some limitations related to the current protocol for diagnosing adult GHD; some of these apply to all diagnostic tests for GHD, while others are specific to individual tests.

Among the general considerations, we must recognize that diagnosing adult GHD poses a challenge for clinicians due to the absence of a singular biological marker. To date, the diagnosis of GHD depends solely on biochemical evidence showing a diminished peak serum GH level in response to one or more GH stimulation tests [1,2,3,4]. At present, no optimal stimulation test exists, and the decision to perform a specific GH stimulation test must consider the reliability of the selected test, its GH thresholds, the accessibility of local resources, and the clinician’s proficiency. Initially, diagnostic cut-offs for various stimulation tests were determined by identifying the minimal response observed in a group of normal subjects [56,57]. Subsequent studies have revisited these diagnostic criteria by comparing the test response under analysis with that of the gold standard test [43,46,58,59], often involving comparisons between patients and normal subjects [42,43,46]. However, all these approaches are susceptible to bias. Specifically, studies comparing the response of patients already diagnosed with the target condition to that of healthy volunteers, even when matched by sex, age, and BMI, risk overestimating the diagnostic accuracy of the identified cut-offs. Consequently, these results may not be applicable in clinical settings. Conversely, studies establishing the diagnostic accuracy of a particular test by comparing it with another diagnostic test may overestimate or underestimate the diagnosis similarly to the gold standard itself.

Another important consideration when interpreting the results of various studies conducted over the years is that the GH specific thresholds proposed in any study are dependent on the assay method employed. In many cases, variability between assay results can exceed 100%, which limits the applicability of assay-specific thresholds in routine clinical practice [60]. Factors contributing to the variation in GH assay results include the heterogeneity of the analyte itself, the accessibility of various calibration standards, and possible interference from other substances such as growth hormone binding protein (GHBP) [61].

Therefore, it is crucial to consider both test- and assay-specific cut-off points to prevent misinterpretation and caution must be exercised before adopting or extrapolating cut-off values from other laboratories [60,62,63].

Among the peculiar limitations of some specific tests currently available for the diagnosis of GHD, we must remember:

1. About ITT:
  (a) It requires close medical supervision, is unpleasant for patients, and can present significant adverse effects such as seizures and altered consciousness due to neuroglycopenia. Additionally, inducing adequate hypoglycemia in obese patients with insulin resistance can be difficult, necessitating the use of higher doses of insulin (0.15–0.2 IU/kg), thereby increasing the risk of delayed hypoglycemia after the test is completed. In this regard, a study has proposed the possibility of infusing a low dose of glucose after achieving the appropriate level of hypoglycemia during the ITT to alleviate patient discomfort and make the test less hazardous, without significantly altering the GH response [64]. However, further studies are needed to consider the implementation of this protocol in clinical practice.
  (b) While the ITT shows high sensitivity, its lack of reproducibility presents another limitation: variations in peak GH responses have been noted in healthy individuals undergoing ITT at different time points [65] and in women across different phases of the menstrual cycle [66].
  (c) We lack normative data based on BMI: we recently proposed new BMI-related cut-offs [59], but our results need further confirmation.
2. About GST:
  (a) It is important to highlight that even the latest studies re-assessing GST thresholds in relation to BMI [49,50,51] have been performed on small cohorts of participants. Therefore, further validation of these findings on a larger population is required.
  (b) Reports on the correlation between peak GH during the GST and age are mixed [46,48,50,51,52,67]. As a result, the application of the same diagnostic cut-offs for the GST across all adult age groups is currently being reconsidered. In this regard, it is important to highlight that a recent study attempted to identify the optimal GH response cut-off to the GST during the transition phase [68], demonstrating that the application of the cut-offs currently used in adults is largely inadequate for this specific stage of life. Moreover, it should be noted that none of the existing GST studies have included elderly patients (age > 70 years), making it difficult to extrapolate findings from younger populations to older ones, where underlying co-morbidities may be present.
  (c) Similar to age, it is still unclear whether the GH response to the GST is influenced by sex [45,50,51,52,67,69]. Furthermore, no study to date has specifically examined the need to adjust the GST cut-offs based on gender.
  (d) The literature presents conflicting data regarding the correlation between peak GH response and factors such as fasting baseline, peak, nadir, or rate of change in blood glucose levels following GST. While some studies have previously reported no significant association between GH response and glucose levels during the GST, the importance of glucose monitoring during GST has recently been emphasized to validate GH stimulation and support clinical decisions in GH deficiency management [70].
3. About Macimorelin:
  (a) Reduced GH responsiveness to all stimulation tests has been observed in individuals with obesity and abdominal adiposity [71,72,73]. Consequently, it is essential to use BMI-related cut-offs for the interpretation of any GH stimulation test. However, it should be considered that, unlike other tests, to date Macimorelin has no specific cut-offs for overweight or obese patients.
  (b) GH secretion normally decreases with age. In patients up to 60 years of age, the diagnostic performance of Macimorelin was comparable to that of the ITT [74]. For those aged between 60 and 65 years, the limited available data do not suggest the necessity for a distinct cut-off point. However, the efficacy of Macimorelin in patients over 65 years remains unconfirmed.
  (c) The safety and efficacy of Macimorelin in children and adolescents under 18 years have not yet been determined. Additionally, the cut-off point for Macimorelin during the transition from late puberty to full adult maturation has not been established.
4. About GHRH + Arginine and GHRH + GHRP-6: Considering the total unavailability of GHRH that has occurred over the past 10 years, these two tests are unfortunately no longer feasible.

## 4. Update on Adult GHD Treatment

### 4.1. When to Treat

One of the mainstays of endocrinology has always been that, if a hormone is deficient, the appropriate therapy for that condition is the substitution the closest possible to the physiological doses and rhythms of endogenous production. This is also true for GHD in adulthood, given that the role of GH on humans is far beyond that of linear growth during pubertal phase.

Current guidelines suggest starting therapy with rhGH at the time of diagnosis in all patients without contraindications and with any signs or symptoms of GHD syndrome in order to improve body composition, muscular capacity, bone health and overall QoL [2,3,4]. In the United Kingdom, impaired baseline QoL using the Assessment of Growth Hormone Deficiency in Adults (AGHDA) questionnaire is a prerequisite to initiate GH substitution [75], while in other countries, QoL questionnaires are not used, which is in accordance with the guidelines. Finally, it must be underlined that in the ESE audit on management of adult GHD in clinical practice, published in 2021, in two-thirds of the participating centers, a low baseline IGF-I level was required to initiate GH therapy, despite guidelines stating that a normal IGF-I does not exclude GHD at any age [39].

### 4.2. How to Treat and Monitor

The mean production of endogenous GH in adulthood is approximately 0.50 mg/24 h (0.25–0.75) [76], the majority of which produced at night, closely related to slow wave sleep phases [77]. However, GH secretion is highly variable in various physiological and pathological conditions and drastically decreases during the normal aging process, falling even below 0.10 mg/24 h in older healthy subjects [78]. Gender, and estrogenic status in particular, also exerts a strong influence on body response to GH, because oestrogens blunt sensitivity to GH particular in the liver, so that women in their fertile phase or that are taking oral oestrogens need more GH to attain the same biological effects [79].

These data are crucially important when setting up a treatment in naïve patients or patients who were previously in therapy. In fact, the weight-based approach adopted in children would seriously overestimate GH requirements if transposed to the adult patient. The main principle of treatment is to begin somewhat low on daily dose and up-titrate to find the maintenance range, as opposed to starting high and down-titrate, but the starting dose is variable depending on clinical parameters, some of which already cited: age, gender, use of oral contraceptives and metabolic status [3].

International guidelines are overall concordant on age clusters of patients requiring differential doses [2,3]:

- Patients transitioning from paediatric care, in whom GHD has been confirmed, generally require the highest dosage: a safe approach could be resuming therapy at 50% the dosage already used in childhood.
- Age < 30 years: usual range is 0.3 to 0.5 mg of rhGH/day.
- Age 30–60 years: 0.2 to 0.3 mg/day.
- Age > 60 years: 0.1 to 0.2 mg/day.

A low starting dosage (0.1–0.2 mg/day) is also recommended in diabetic and/or obese patients and in women with previously diagnosed gestational diabetes mellitus (GDM), due to the early tendency of rhGH to oppose insulin action. Women taking oral contraceptive pills frequently need to start from the highest range for their age group, or possibly switch to transdermal oestrogens formulations, the latter being less impactful on GH antagonism due to lack of first-hepatic passage, even though data are lacking on high dose transdermal formulations [80,81].

The classic therapy is given subcutaneously in the evening to mimic as best as possible the circadian rhythm of the somatotropic axis, although different regimens have been proposed mainly to improve patients’ compliance, keeping the same total weekly dose. A three injections per week scheme has been observed to be equally effective on main clinical endpoints and IGF-I normalisation [82,83].

As a parenteral therapy, injection sites should vary to limit local lipoatrophy that could alter drug absorption.

Regarding various commercially available rhGH formulations, none of them is recommended over the others in terms of efficacy, but different preparations could be best suited for a specific patient’s needs over another; some variables to take into account could be a true needle phobia, that directs the choice towards a jet-injector system, even if not totally pain-free, or the logistic preference of the patient over preparations that require or do not require refrigeration [84]; also, the range of possibilities could be limited due to national or regional directive.

Once started, monitoring is suggested at 4 to 8 weeks intervals until the appropriate maintenance dosing is found [2,3,4]. The optimum goal to reach has not univocally been identified. There is no single biochemical marker that strongly correlates with clinical endpoints, such as body composition changes or QoL [85]. To this date, IGF-I remains the most widely used biomarker for assessing GH exposure; there are two main critical points of this serum marker:

- A different target the various guidelines proposed throughout the years, being “the age-adjusted reference range provided by the laboratory” for AACE 2019 guideline [3], “the upper half of that range” for ES 2011 guideline [2], “below the upper limit of normal” for ES 2016 guideline [4].
- As stated above, IGF-I alone is not a diagnostic tool for GHD; a vast portion of patients presents with in-range IGF-I values, and a non-negligible percentage of patients could present with above-median values of IGF-I for age and sex-matched reference range, possibly making this parameter less informative during follow-up [86,87].

The other main goal is reaching the chosen IGF-I target without side effects, or the highest-tolerated dose if target IGF-I is associated with unpleasant symptoms [2,3].

Parallel issues to consider are other eventual pituitary deficits, as GH exert strong interactions with other hormones [4]. We already disclosed the main interaction between GH and oestrogens, but it is important to outline any criticalities regarding other endocrine axes and, in particular:

- GH limits the conversion from cortisone to cortisol, so that patients already in therapy could require higher doses, or GH could unmask a latent cortisol deficit in apparently healthy individuals [88].
- GH also increase peripheral clearance of free tiroxine (fT4); in an analogue manner as for cortisol, patients taking levothyroxine could require higher doses after rhGH initiation, or a central hypothyroidism could be unmasked if previously undiagnosed [89].

Thus, it is recommended to evaluate thyroid functionality tests and basal cortisol after rhGH initiation and possibly after any dose increase; if necessary, perform a dynamic test for hypothalamus–pituitary–adrenal axis integrity [3].

After reaching goal targets for treatment, guidelines suggest follow-up visits at 6- to 12-month intervals [2,3]. Apart from IGF-I and clinical monitoring, there are other parameters that should be looked up. An evaluation of metabolic serum markers, such as fasting glucose, HbA1c and lipids profile should be performed at least annually, both for assessing any amelioration following treatment and for detecting any worsening of the glycaemic status, as furtherly discussed in the next paragraph. Anthropometric values such as weight, BMI, and waist and hip circumference should be noted at every visit, as well as any possible side effects related to the therapy, as long as the general conditions.

Performing a Dual-Energy X-ray Absorptiometry (DEXA) scan is suggested every 18–24 months if already abnormal at baseline evaluation, as in common clinical practice.

If any dose variation is required, the approach is analogous to the titration phase.

As rhGH tends to manifest its beneficial effects after at least 6 months of treatment, it is general indication to wait at least one year before considering to stop therapy in a patient who doesn’t show signs of improvement [2,3]. If patients opt to discontinue rhGH replacement therapy, a follow-up appointment after 6 months is advised, as some may decide to restart treatment upon recognizing, in hindsight, that they experienced enhanced well-being during therapy [3]. Regarding patients in whom the therapy appeared to be beneficial, there is no general consensus on when to stop; data are lacking on subjects > 80 years old, but there is no reason to stop if a cautious dosage is used and the patient is willing to prosecute.

**Figure 1:**
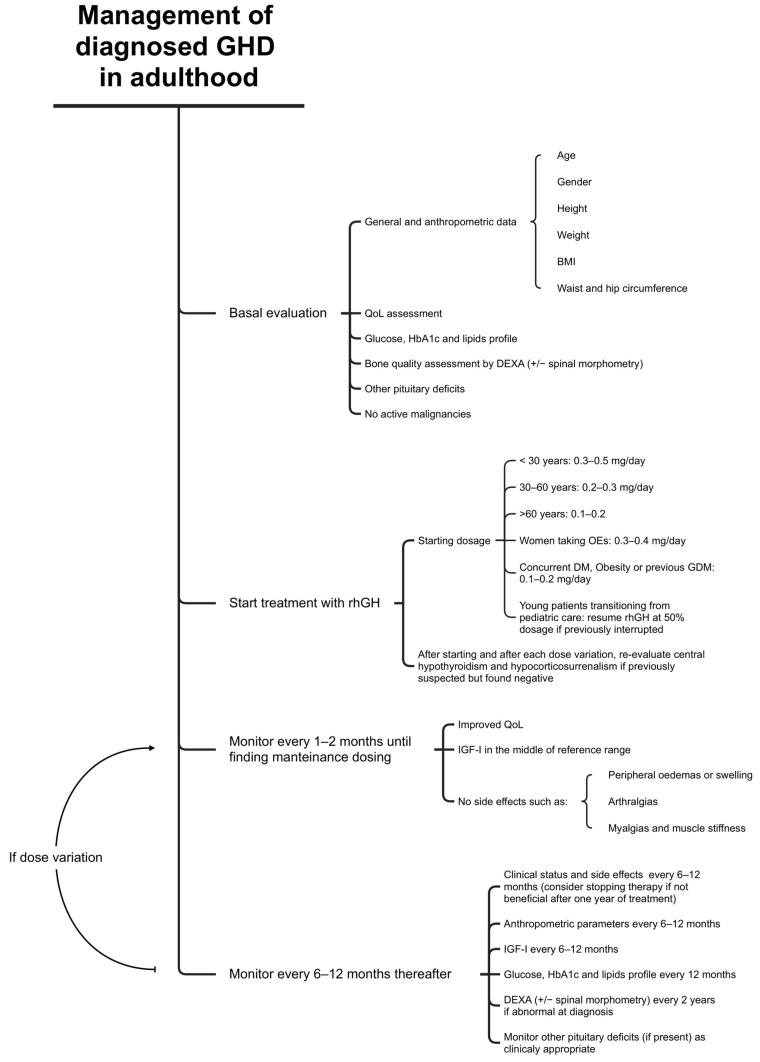
Management of GHD in adults (original figure). BMI, body mass index; DEXA, Dual-Energy X-ray Absorptiometry; DM, diabetes mellitus; GDM, gestational diabetes mellitus; Oes, oral estrogen; QoL, quality of life; rhGH, recombinant human GH.

A schematic synthesis of the management of GHD in the adult has been provided in Figure 1.

### 4.3. Drawbacks of rhGH

As many replacement therapies, treatment with rhGH is generally well tolerated, even though it has its own sharp edges.

#### 4.3.1. A Matter of Compliance

As hinted in the last paragraph, compliance is a major issue in GHD substitution therapy, with reduction in adherence varying from 10 to 30% [84,90], for at least two reasons: as a parenteral treatment, it could be perceived as burdensome by some patients; furthermore, GHD bearing a vague and subtle array of symptoms, it is different for the patient to feel the clinical benefit, more so if we consider that improvements typically start to manifest after 6 months or more of treatment [84]; two possible aggravating factors in the subgroup of multiple pituitary deficit patients are both the necessity to withstand a greater number of medications, and the more evident and acute impact that other deficits have on the general perception of well-being from the patient’s perspective.

Besides the classical daily regimen, different schedules have been tried to enhance patient adherence; surprisingly, the 7 days regimen appeared to be better followed than a 5 or 6 days per week in an Italian population, possibly due to either a confusion factor or the underestimation of therapy relevance in relation to day-skipping [90]. A promising alternative is 3-day regimen, possibly on alternate days, that alleviates the burden of everyday injection, still keeping a fixed schedule, and already proved itself to be comparable in efficacy to the standard scheme [82].

#### 4.3.2. Side Effects

Acute side effects related to rhGH therapy primarily related to the exaggeration of physiological actions of GH such as sodium and water retention [2,3,4].

Patients with GHD have significantly less extracellular volume, such as a good substitution regimen usually leads to a ~1 kg gain in weight after one year of treatment [91]; at the opposite of the spectrum, excessive doses of rhGH could lead to peripheral oedema and swelling of soft tissues, leading to paraesthesia, arthralgia, myalgia and muscle stiffness. These kind of side effects were more commonly seen when weight-based dosing was used, now they are a signal of likely overdosing and usually recede after lowering dosage or after a brief pause from therapy, starting lower at resumption [2,3].

#### 4.3.3. Contraindications and Long-Term Concerns

The anabolic effect of GH and IGF-I and their role in the promotion of growth in solid tissues has always been a concern regarding a theoretical oncological burden. To this date, there is no evidence that rhGH increases the risk for solid tumours or for recurrence of pituitary adenomas or craniopharyngiomas [92,93,94,95].

Nevertheless, the presence of an active malignancy remains at the present time the only true contraindication to beginning therapy with rhGH [2,3]. As for adult patients with anamnestic evidence of cancer that are currently on remission, guidelines empirically suggest a safe interval of at least 5 years free of disease before considering a low-dose replacement regimen [3]. For treated pituitary adenomas and craniopharyngiomas, there is still debate if therapy could be started right away after correcting for any other pituitary deficit, or if a safer waiting time of 12 months should be advised [96]. Caution is also advised in patients with monogenic disease known to bear significant oncological risk.

Patients with GHD present themselves with a metabolic status similar to the classic metabolic syndrome, with altered body composition and impaired glucose metabolism per se, increasing their basal risk of developing DM [2,3]. The natural opposing action of GH on insulin has led to ponder if rhGH replacement therapy could potentially worsen glycaemic status of these patients. For many years has been known that a tendency to insulin resistance arise at the beginning of rhGH therapy [97]; the main physiopathological line of reasoning for pursuing treatment in this regard has been a favourable change in body composition after 1 to 2 years of treatment, that could potentially rebalance the net effect of insulin sensitivity. To this date, the true net long-term balance of rhGH on insulin sensitivity has not been cleared, with data from different studies showing conflicting results. Diabetes by itself does not represent a contraindication to starting therapy, but it is suggested to begin with low dosage and, if necessary, achieve good glycaemic control before attempting treatment with rhGH [2,3]. Long term data is still required to clarify this matter, but the data trend shifts towards the hypothesis that the overall effect is beneficial on insulin sensitivity and that rhGH replacement could at least partially prevent the physiological advancing of insulin resistance in aging [98,99]; still, close monitoring of glucose metabolism is advised in GHD patients, naïve or during replacement therapy. If DM happens to develop, management is no different from that of the general population. It could be useful to keep in mind that fasting morning glucose (FMG) could be rise still within normal limits after starting therapy, not to be interpreted as a direct sign of insulin sensitivity worsening, as evening administration of rhGH could enhance morning levels without a 24 h deterioration of glycaemic control but, on the contrary, a better overall glycaemic curve [97]; thus, it is recommended to measure HbA1c together with FMG for a better representation of glycaemic status.

#### 4.3.4. Grey Areas in Adult GHD Treatment

More than 30 years after the initial use of rhGH in the treatment of adult GHD, this replacement therapy continues to pose several challenges and uncertainties for clinical endocrinologists [39]. Although numerous studies and meta-analyses have elucidated the beneficial effects of rhGH therapy, they have also introduced new concerns over time (Table 2). For instance, while it is well-established that rhGH therapy reduces fat mass, increases lean body mass, lowers total and LDL cholesterol levels, and decreases diastolic blood pressure [22,23,24], there are also studies that have shown increases in BMI, waist circumference, waist-to-hip ratio, blood glucose, and insulin levels, along with a reduction in insulin sensitivity, leading to a higher prevalence of metabolic syndrome during rhGH therapy [3,22,25]. If left untreated, adult GHD can increase the risk of bone fractures, obesity, DM, dyslipidaemia, and cardiovascular and cerebrovascular diseases [1,2,3,4], although not all studies agree [100]. Additionally, prior research has suggested that hypopituitarism is associated with increased mortality [13,18], partly attributable to GHD [101]. However, to date, no study has conclusively demonstrated a normalization of this elevated mortality risk with rhGH therapy.

Patients with GHD also have a 2- to 5-fold higher risk of fractures compared to the general population, a risk that is particularly relevant in adult GHD [105]. Replacement therapy with rhGH is strongly associated with increased BMD and a lower incidence of fractures, but the evidence supporting a reduced fracture risk is much less robust [103,106,107]. Furthermore, BMD and other commonly used bone markers do not strongly correlate with fracture risk in this patient population, making the follow-up of this particular co-morbidity in individual patients somewhat misleading [108]. The presence of pre-existing vertebral fractures is a strong indicator of skeletal fragility [103], which is why incorporating spinal morphometry alongside DEXA scans may be useful to better detect disease progression.

Data on fertility and pregnancy is scarce, and for this reason rhGH is currently not approved for use during pregnancy. Previous studies tried to answer the question whether a good replacement regimen could affects gestational outcomes; neither prosecution of therapy nor untreated GHD seemed to significantly alter the pregnancy course [109,110], but there is small evidence that rhGH could augment fertility prior to conception [111]. As the normal circadian secretion is progressively overcome by continuous placental production, other researchers used a tapered scheme in eight GHD women in which the physiological rhGH dose was given during the first trimester and then gradually reduced, until completely stopping by the third trimester, without observing any major complications [112]. Despite its physiopathological rationale, the lack of solid evidence on this topic currently precludes the routine use of rhGH for conception or its continued administration during pregnancy in women with GHD [3].

Lastly, cost-effectiveness studies evaluating the use of rhGH in adults with GHD remain scarce. While robust safety data exist, with approximately 200,000 patients and over 500,000 patient-years documented in pharmaceutical company-sponsored post-marketing surveillance studies [1,2,3,4], these findings are limited by methodological constraints. The open-label design introduces intrinsic biases in cohort monitoring, as outcomes rely heavily on physician-reported assessments to identify potential ‘GH-related’ events. Additionally, these studies may have evaluated varying GH dosages and/or patient characteristics throughout the study period, are subject to time limitations, often lack adequate control groups, and may miss adverse events that emerge after treatment. Furthermore, the involvement of the sponsoring company may undermine the ability to derive truly comprehensive and reliable conclusions.

## 5. Future Perspectives

Considering the significant cost of rhGH treatment and its potential long-term risks, it is essential to establish an accurate diagnosis to ensure that GH replacement therapy is administered solely to adults who genuinely have GHD. Given the limitations of the GH stimulation tests discussed in the previous section, it is advisable to validate each stimulation test specifically in subjects with hypothalamic-pituitary disease, including both those with GHD (cases) and those without (controls), from a clinical perspective. If the diagnostic threshold for a particular test is set too high—such as when comparing patients definitively diagnosed with GHD to healthy individuals—the identified patients may not exhibit severe GHD, leading to potentially less severe clinical consequences. Consequently, the long-term outcomes for these patients may differ from those with severe GHD, possibly resulting in a diminished perceived benefit from GH replacement therapy. Additionally, a substantial number of such patients could undermine the accuracy of outcome data derived from post-marketing surveillance studies [113].

It is important to note that the proportion of patients showing low GH responses to provocative tests increases with the number of other pituitary hormone deficiencies. Several studies involving panhypopituitary patients have indicated that, under certain circumstances, GH stimulation tests may be unnecessary for diagnosing GHD [1,2,3,4]. Therefore, the remaining pituitary function can serve as a valid gold standard for determining the presence or absence of GHD. Recently, we utilized this approach to re-evaluate the BMI-related cut-offs for the GHRH + Arginine test, highlighting the need to re-assess current cut-offs [42] to reduce false positive diagnoses of GHD [113].

Therefore, in general terms and regardless of the stimulation test considered, the use of a clinical gold standard, such as residual pituitary function, would be desirable in future studies aimed at redefining the optimal cut-off for the diagnosis of adult GHD.

Another approach to improve the diagnosis of adult GHD could be the development of clinical scores to integrate with the results of the stimulation test. Such an approach has already been developed for other pathological conditions [114], and some attempts have also been made in the diagnosis of GHD [115,116].

As for the specific diagnostic tests, it would be appropriate to:

1. About GST: given the conflicting data regarding a potential association between age or gender and the GH response to GST, future studies will need to specifically assess whether diagnostic cut-offs should be adjusted based on these factors [117]. The evaluation and validation of specific GST diagnostic thresholds are particularly urgent in older populations and in certain pathological conditions, such as Prader–Willi syndrome, which remain underresearched [118].
2. Additionally, we recently observed significant hypokalemia in over 50% of subjects tested, as an unexpected side effect of intranasal glucagon administration [119]. To date, no cases of hypokalemia have been reported with intramuscular or subcutaneous glucagon administration. However, it remains unclear whether this reflects a higher safety profile of these administration routes compared to the intranasal route or simply the lack of potassium monitoring during GST administration.

About Macimorelin: the use of the Macimorelin test has occasionally been associated with QT interval prolongation [53], leading to the inclusion of a warning in the summary of product characteristics (https://www.ema.europa.eu/en/medicines/human/EPAR/ghryvelin) (accessed on 10 September 2024). Furthermore, considering that several case reports [120,121,122,123,124,125,126,127,128,129,130,131,132,133,134,135] suggest an association between hypopituitarism and an acquired form of QT prolongation, further safety studies on the use of Macimorelin in patients already at higher risk of presenting with QT prolongation are essential.

Additionally, there is currently insufficient data available regarding potential BMI-dependent cut-offs for the diagnosis of adult GHD with Macimorelin, and even fewer data are available regarding its use during the transition phase.

Concerning biochemical monitoring during rhGH therapy, to date, serum IGF-I remains a valuable and convenient tool to detect under and over-replacements during rhGH treatment [1,2,3,4,136], but it has a poor relationship with clinical efficacy endpoints. Therefore, there is a pressing need for reliable biomarkers to assess responses to GH replacement therapy [137,138]. Moreover, regarding IGF-I levels assessment, it must be emphasized that there is no agreement on the IGF-I levels to be achieved during replacement therapy as it has been recently demonstrated by the ESE audit on management of adult GHD [39]; in this study, the target IGF-I concentration to be reached during treatment was a value within the normal range in 40%, within the mid-normal range in 26%, at a high-normal range in 14%, and at a low-normal range in 3% of the participating centers. Finally, it would be advisable to better define how to manage and monitor those patients who, even before initiating rhGH therapy, exhibit IGF-I levels well within the normal range for their age group, which is generally the biochemical target suggested by current guidelines.

Regarding rhGH replacement therapy, it is still administered daily via subcutaneous injection and potentially for life. Although it is effective in improving patient QoL, it is often associated with suboptimal treatment adherence, as demonstrated by several studies [90,139]. Nonadherence to rhGH therapy is a common issue and negatively affects treatment efficacy, resulting in increased healthcare costs [140]. Enhancing compliance and persistence in managing GHD is a key priority. The frequency of injections is considered one of the main factors contributing to nonadherence in adults with GHD; therefore, a number of companies have been developing long-acting GH preparations aiming to lower the frequency dosing schedule; this would be potentially less burdensome to patients and may improve adherence to treatment. Since long-acting GH preparations will likely gain approval by regulatory authorities in the coming years, it will be essential to closely monitor these products, particularly regarding their long-term safety, as well as to confirm the hypothesized improvement in treatment adherence with their use, which does not seem to be supported by all experiences [141].

## 6. Conclusions

This review advances the understanding of adult GHD management by highlighting the importance of personalized approaches in both diagnosis and treatment. From a diagnostic standpoint, the synthesis of existing literature underscores the need to standardize and validate current and future protocols using a clinical gold standard. Such an approach would reduce false positives, optimize healthcare costs, and provide a clearer assessment of rhGH replacement therapy’s effectiveness in lowering cardiovascular risk and mortality. Regarding therapeutic efficacy, patient compliance and adherence remain major limitations. Future research should explore whether the introduction of long-acting formulations, which could enhance adherence, leads to more substantial and consistent improvements in both safety and efficacy outcomes.

## Figures and Tables

**Table 1:** Diagnostic tests for adult growth hormone deficiency.

	Procedure	Diagnostic Cut-Offs for GHD	Aderse Events	Contraindications	Guidelines
**ITT**	- Insulin 0.05–0.15 IU/kg i.v. - Blood sample for GH and glucose at −30, 0, 30, 60, 120 min	Glucose should drop < 40 mg/dL GHRS 2007: GH < 3 μg/L [40] ES 2011: GH < 5.1 µg/L [41] ES 2016: GH ≤ 3–5 μg/L [40,41] AACE 2019: GH ≤ 5 μg/L [41]	Severe hypoglycemia with patient discomfort, seizure and altered consciousness	- Pregnancy - Age > 65 years - Epilepsy - CVD history	GHRS 2007 [1] ES 2011 [2] ES 2016 [4] AACE 2019 [3]
**GHRH + Arginine**	- GHRH 1–44 1 µg/kg i.v. + infusion of Arginine HCl 0.5 g/kg (max 30 g). - Blood sample for GH at 30–45–60 min	GHRS 2007: [42] <11.0 µg/L if BMI < 25 kg/m^2^ <8 µg/L if BMI 25–30 kg/m^2^ <4.0 µg/L if BMI > 30 kg/m^2^ ES 2011: GH < 4.1 µg/L [41] It would be reasonable to use different cut points according to BMI. ES 2016: GH ≤ 4 μg/L [41], but cut-offs for GH response should be correlated to BMI.	Flushing, nausea, taste, and smell alterations	- Chronic renal failure	GHRS 2007 [1] ES 2011 [2] ES 2016 [4]
**GHRH + GHRP-6**	- GHRH 1–44 1 µg/kg i.v. + GHRP-6 1 µg/kg i.v. - Blood sample for GH at 0–15–30 min	GHRS 2007: <10 µg/L if BMI ≤ 35 kg/m^2^ [43] <5 µg/L if BMI > 35 kg/m^2^ [44]	Flushing	- None	GHRS 2007 [1]
**GST**	- Glucagon 1–1.5 mg i.m. - Blood sample for GH and glucose at 0–30–60–90–120–150–180–210–240 min	GHRS 2007: GH < 3 μg/L [45,46] ES 2011: GH < 2.5–3 μg/L [45,46,47] ES 2016: GH ≤ 3 μg/L [45,46], but cut-offs for GH response should be correlated to BMI. AACE 2019 [48,49,50,51,52]: ≤3 µg/L if BMI < 25 kg/m^2^ or BMI 25–30 kg/m^2^ with high pre-test probability ≤1 µg/L if BMI 25–30 kg/m^2^ with low pre-test probability or if BMI > 30 kg/m^2^	Nausea, vomiting, late hypoglycemia	- Fasting hyperglicemia - Insulinoma	GHRS 2007 [1] ES 2011 [2] ES 2016 [4] AACE 2019 [3]
**MACI**	- Macimorelin 0.5 mg/kg in 1 mL/kg orally - Blood sample for GH at 30–45–60–90 min	AACE 2019: ≤2.8 µg/L [53]	Dysgeusia	- Long QT syndrome	AACE 2019 [3]

GHRH, growth hormone releasing hormone; ITT, insulin tolerance test; GHRP-6, growth hormone releasing peptide 6; GST, glucagon stimulation test; MACI, macimorelin; i.v., intravenous; i.m., intramuscular; GH, growth hormone; BMI, body mass index; GHRS, Growth Hormone Research Society; ES, Endocrine Society; AACE, American Association of Clinical Endocrinology.

**Table 2:** Lights and shadows of rhGH therapy in the adult.

Patient’s Perspective		CV Risk		Metabolic Status	
- Improved QoL [75]	- Non-optimal compliance could mitigate improvements	- Reduction in LDL [22,23] - Reduction in DBP [22] - Improved LVF [26] - Reduction in CRP [102]	- Possible worsening of glycaemic status [97]	- Improved body composition [23] - Improved exercise capacity [32]	- Initial insulin-resistance [97] - Possible long-term risk of DM development
**Bone health**		**Oncological burden**		**Mortality**	
- Improved BMD [31] - Small evidence of fracture risk reduction [103]	- No optimal follow-up methods - More evidence needed on fracture risk	- No data showing increased oncological risk	- Physiopathological rationale on tumour risk - Contraindication during active malignancy - No optimal timing for starting treatment after cancer remission	- Lower incidence of myocardial infarction [104]	- No data showing reduced overall mortality

BMD, bone mineral density; CV, cardiovascular; DBP, diastolic blood pressure; DM, diabetes mellitus; LDL, low-density cholesterol; LVF, left ventricular function; CRP, C-reactive protein; QoL, quality of life.

## Data Availability

No new data were created or analyzed in this study. Data sharing is not applicable to this article.
